# Supplementary material for: Development of an In-House EphA2 ELISA for Human Serum and Measurement of Circulating Levels of EphA2 in Hypertensive Patients with Renal Dysfunction
Source: Diagnostics (Basel). 2022 Dec 2;12(12):3023. doi: 10.3390/diagnostics12123023 (PMC9776842; doi:10.3390/diagnostics12123023)
Supplement: Supplementary file 1 [file diagnostics-12-03023-s001.zip › diagnostics-2042047-supplementary.pdf]

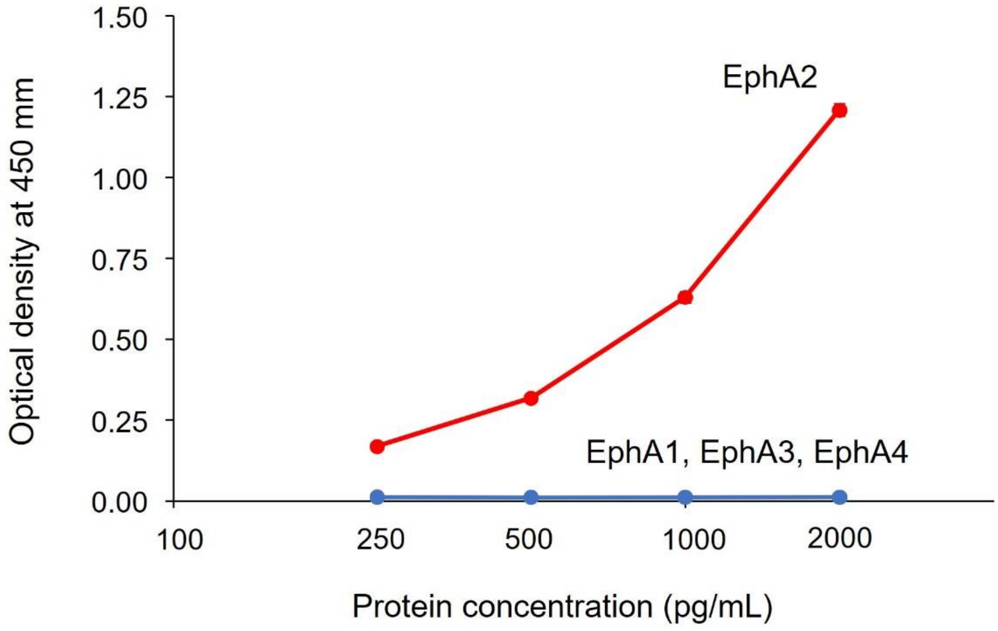

Figure S1. Specificity for EphA2. No cross-reactivity was noted with other Eph receptors (i.e., EphA1, EphA3, and EphA4).

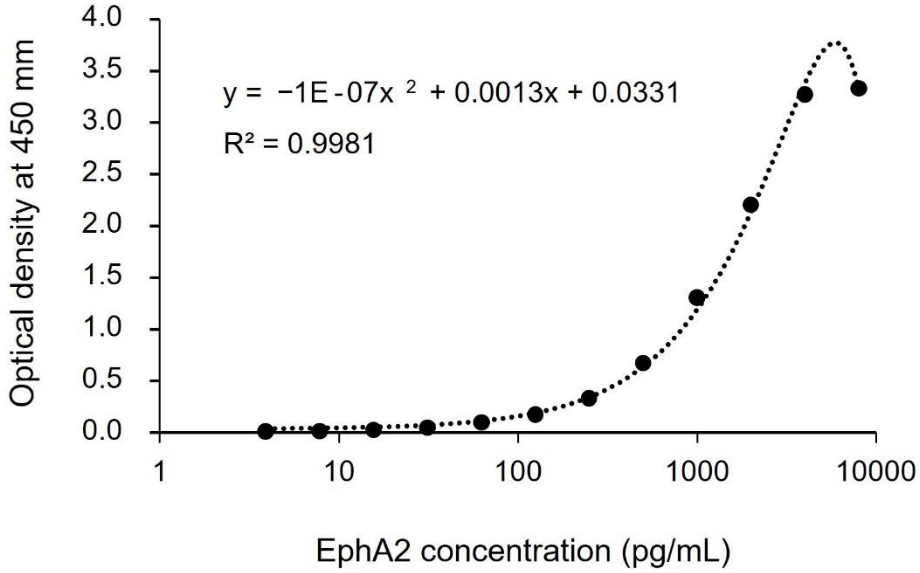

Figure S2. Calibration curve of EphA2 using a second-degree polynomial. A typical calibration curve is depicted across the concentration range (3.9–4000 pg/mL). The hook effect was observed at an EphA2 concentration of 8000 pg/mL.

Table S1. Spike and recovery test.

Serum EphA2 Level	Spike Level			Average % Recovery
	Low (200 pg/mL)	Medium (600 pg/mL)	High (1800 pg/mL)	
High (n = 2)	79	81	80	80
Medium (n = 2)	79	81	86	82
Low (n = 2)	87	97	111	98
Average % recovery	82	86	92	87

EphA2 level: high (1500–4000 pg/mL), medium (500–1500 pg/mL), and low (<500 pg/mL).

Table S2. Dilution linearity test.

Serum EphA2 Level	Dilution Factor (-Fold)				Average % Recovery
	2	4	8	16	
Very high (n = 4)	101	97	103	103	101
High (n = 4)	85	91	101	101	94
Medium (n = 4)	89	93	99	108	97
Low (n = 4)	117	119	132	NA	123
Average % recovery	98	100	109	104	103

Serum EphA2 level: very high (2000–4000 pg/mL), high (1000–2000 pg/mL), medium (500–1000 pg/mL), low (<500 pg/mL).

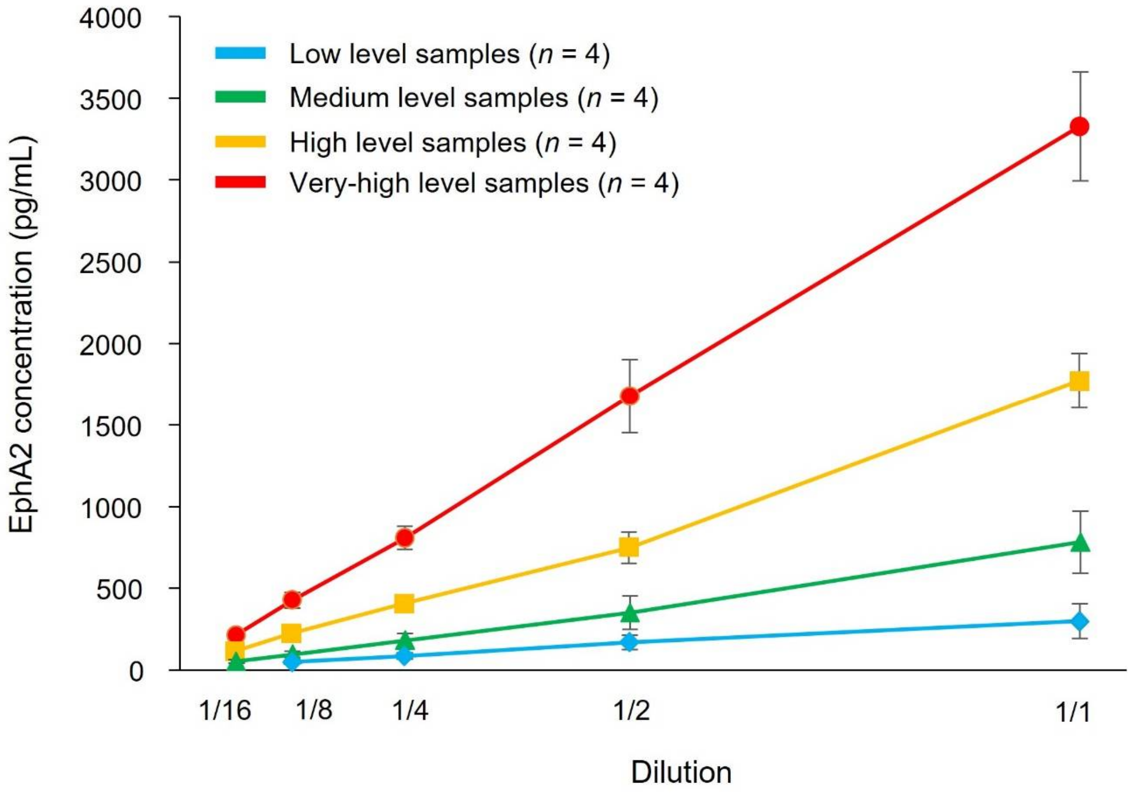

Figure S3. Linearity. Dilution linearity of EphA2 was assessed with two-, four-, eight- and 16-fold dilutions. The quantitation of EphA2 at all tested dilutions was accurate and precise.

Table S3. Precision test.

Serum EphA2 level	Intra-assay CV (%)	Inter-assay CV (%)
Very high (*n* = 4)	2.0	1.7
High (*n* = 4)	2.9	3.0
Medium (*n* = 4)	2.8	2.5
Low (*n* = 4)	3.3	6.7
Average	2.8	3.5

Serum EphA2 level: very high (2000–4000 pg/mL), high (1000–2000 pg/mL), medium (500–1000 pg/mL), and low (<500 pg/mL).

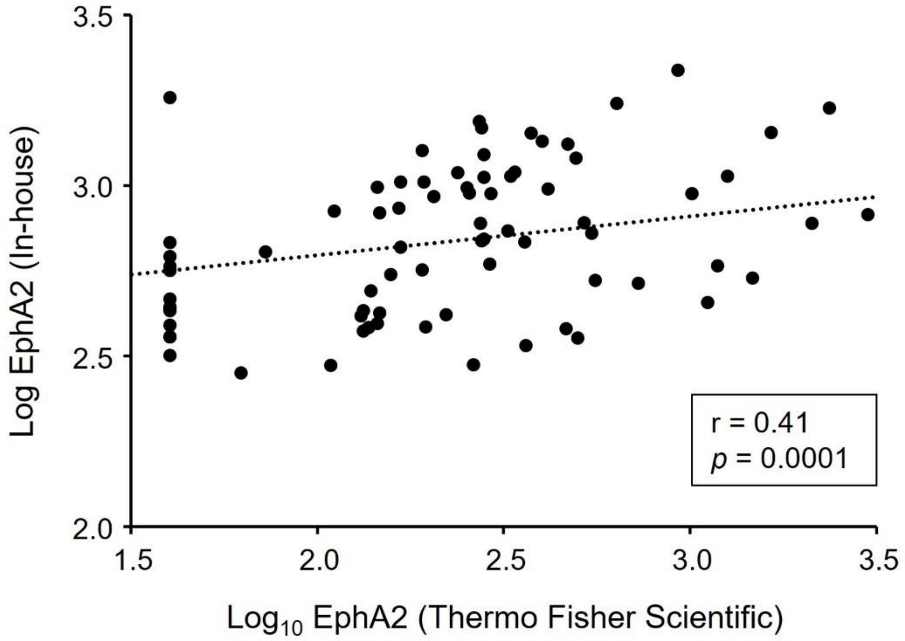

Figure S4. The correlation of EphA2 results between the assays performed with the in-house ELISA and ELISA kit from Thermo Fisher Scientific. The concentrations of EphA2 measured with the novel in-house ELISA correlated significantly but poorly with the EphA2 concentrations measured with the ELISA from ThermoFisher Scientific.

Table S4. Comparison of novel in-house EphA2 ELISA with a Thermo Fisher Scientific ELISA in the study patients.

	In-house	Thermo Fisher Scientific
Analytical sensitivity (pg/mL)	29.5	80 [a]
Assay range (pg/mL)	31.3–2000	82–20,000 [a]
Intra-assay CV (%)	2.8	<10 [a]
Inter-assay CV (%)	3.5	<12 [a]
Detectability (%)	100.0	83.8
EphA2 (pg/mL)	682 (434, 1006)	252 (131, 464)

[a] Data from the ThermoFisher Scientific product information sheet.
